# Patients and Caregivers Leveraging AI to Improve Their Health Care Journey: Case Study and Lessons Learned

**DOI:** 10.2196/69790

**Published:** 2026-02-20

**Authors:** Mary Beth Schoening, Dustin Cotliar

**Affiliations:** 1 RampUp Health, South Dartmouth, MA, United States; 2 South Shore Health, Weymouth, MA, United States

**Keywords:** artificial intelligence, caregiver AI, care journey, medical information translation, patient-physician relationship, healthcare navigation, large language models, medical documentation, patient education, participatory medicine, healthcare communication, health literacy, LLM, large language model, patient engagement, patient activation, patient learning, patient experience, shared decision making, self efficacy, patient efficacy, generative AI, patient AI, patient friendly, patient empowerment

## Abstract

Artificial intelligence (AI) is increasingly integrated into everyday life. Yet in health care, patients and families are challenged to understand how AI may be helpful. As a result, real-world patient stories remain scarce. Generative AI can serve as a learning partner to help patients interpret complex medical information, prepare for appointments, and navigate care decisions. A case study is presented from the perspective of a caregiver and a clinician colleague, describing how one family used generative AI (ChatGPT; OpenAI) to better understand test results, possible diagnoses and treatments, prepare for visits, and summarize and share information with an extended care team. This paper also shares tips and lessons learned with others navigating similar health care challenges. A first-hand account of family interactions with ChatGPT is described during a period between diagnostic imaging and surgical consultation. Real-world use of AI by a caregiver is showcased, including strategies used to understand and summarize health record data, querying AI using medical documents, and resulting actions taken by the family. Using the case study as a springboard, the authors provide a separate section to share lessons learned for patients and caregivers in their use of AI. The family reported benefits of AI, including the ability to comprehend health information by translating medical records into patient-friendly language; to emotionally process and prepare for visits; to research diagnoses and treatments; to streamline communication with care teams by using concise patient summaries; and to feel more empowered to take timely, informed action. Generative AI can serve as a valuable companion tool for patients and caregivers navigating complex medical information. By translating results, providing education about diagnoses and treatment options, and helping prepare for visits, AI may reduce care delivery delays and raise family confidence in decision-making. However, limitations exist, and patients and caregivers need to validate AI output to ensure accuracy and privacy.

## Case Study of Patient and Caregiver Use of Artificial Intelligence

### Introduction

“Over the course of a few weeks, he went from completing a 3-day golf tournament to being unable to walk and needing to crawl up the stairs to go to bed.”

This dramatic decline was due to RL’s lower back pain, which was observed by MBS, the patient’s spouse, and the primary author of this paper. The co-author (DC) is a colleague of MBS in patient advocacy and artificial intelligence (AI) work and was not clinically involved in this case. MBS’s experience as a caregiver and patient advocate offers unique insight into how AI can transform the patient experience. Her systematic approach to using AI tools not only improved her husband’s care experience but also demonstrated the practical implementation of participatory medicine principles. As she notes:

The ability to understand complex medical information immediately, rather than anxiously waiting, was a game changer, allowing us to act more quickly.

[MBS]

This paper presents a case study from the perspective of a family caregiver who explored how AI can support care by improving the interpretation of medical test results, clarifying potential diagnoses and treatment options, and facilitating more effective care navigation. The case study demonstrates practical ways patients and caregivers can apply AI tools to translate medical information into plain language, increase understanding of conditions and treatments, and summarize health data to prepare for specialist visits.

In addition to the case study, the paper expands on key insights and lessons learned—including additional patient use cases for AI, strategies to address its limitations, highlights of current AI tools, and opportunities for future patient-centered innovation (Section 2).

### The Challenge

The patient, RL, developed intense back pain over a short time period that quickly prevented him from standing or walking. In such moments, patients and their families must rapidly navigate complex medical information, understand treatment options, and make critical decisions—all while dealing with the emotional impact of a rapid health decline.

Today’s health care system gives patients unprecedented access to their medical information through online portals, test results, and clinical notes [1]. However, this access often introduces new challenges. While patients can view their magnetic resonance imaging (MRI) results within hours of a scan, the technical language and medical terminology can make these records more confusing than informative [2]. Limited appointment availability and delays between receiving results and seeing specialists further compound these difficulties [3]. According to a recent study, it now takes an average of 31 days to schedule a physician appointment in 15 of the largest US metropolitan areas—and in the city featured in this case study, the average wait is 65 days [4]. This time lag leaves patients struggling to understand crucial information about their health.

### One Solution: Using AI as a Learning Partner

Artificial intelligence, particularly large language models (LLMs) like ChatGPT (OpenAI), has emerged as an unexpected ally for patients navigating these challenges. In MBS’s words:

These AI tools offered me immediate, around-the-clock assistance in translating complex medical information into language I could understand, when I needed it. That really helped us prepare for appointments, gave us a chance to digest the information, and allowed us to make time-sensitive decisions about my husband’s care.

### Patient AI Journey

MBS discovered that starting with AI to translate MRI results into patient-friendly language and then gradually conducting deeper research led to a greater understanding of the situation. By taking things step by step over several days—each new question adding to her understanding—she steadily built her knowledge and was able to take informed action at each step.

**Figure 1.**
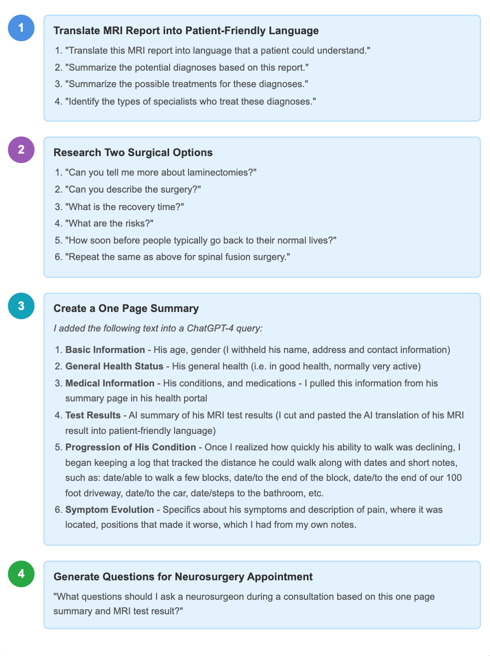
Approach used with generative artificial intelligence by the family caregiver in the case presented. MRI: magnetic resonance imaging; AI: artificial intelligence.

Figure 1 shows the sequence that MBS followed, along with the topics and questions used in the AI conversations. Keep in mind, inputs and questions posed to AI can be combined into a single sentence, or can be part of an iterative conversational flow with the AI chatbot. Patients can type in questions in the same way one would ask a doctor or a friend.

This case shows how generative AI can act as a learning partner for patients and families, helping them make sense of complex medical information through a step-by-step “just-in-time” approach to learning (Figure 1). Instead of having either too little information or being overwhelmed with information all at once, they were able to ask questions and build understanding gradually—learning only what was relevant at each stage of their journey. This reflects the idea of situated learning [5,6], where people learn most effectively when information is connected to real-life needs. It also demonstrates the concept of distributed cognition [7], in which tools like AI extend a person’s ability to think and act.

By engaging with information only when it became meaningful to them, the family strengthened their sense of understanding and control—a pattern long recognized in patient empowerment research [8] and a concept referred to as patient activation, which is linked with improved health outcomes [9].

#### Step 1: Using ChatGPT to Understand MRI Results

When RL received his MRI results through the patient portal, the technical language made it difficult to understand the findings—let alone the implications. Rather than waiting anxiously for the upcoming specialist appointment, MBS turned to ChatGPT for assistance.

First, some background on the AI tool chosen: ChatGPT is one option in the “generative AI” category. It is free to use (with an account) and can be easily found by typing “ChatGPT” into a browser search bar on a mobile device or computer. Once in ChatGPT, users see an empty text field with the words “Ask me anything.” A user can paste text into that field and/or ask questions. Generative AI takes your questions, reviews large sets of data, and then creates new text in response.

ChatGPT is considered a large language model (LLM), a type of generative AI, because it learns patterns from large datasets and generates new output, within the context of the questions you’ve asked. Other forms of AI are “non-generative” and work to analyze or classify information from existing data but do not create new outputs. Nongenerative AI can, for example, recognize patterns in a mammogram and predict “normal” versus “possible tumor.” Generative AI, using an LLM like ChatGPT, is more like a writer, while nongenerative AI is more like a judge. MBS outlines how she used ChatGPT:

When we accessed my husband’s MRI report from the patient portal, we found it difficult to understand because it was written for clinicians. I decided to see how ChatGPT could help us. I copied and pasted the MRI report text, with name and personal information removed, into ChatGPT and asked it the following questions:

1. Translate this MRI report into a language that a patient could understand.
2. Summarize the potential diagnoses based on this report.
3. Summarize the possible treatments for these diagnoses.
4. Identify the types of specialists who treat these diagnoses.

In literally seconds, ChatGPT came back with results from this query with information that was much easier to understand. This approach benefited MBS and RL in a number of ways:

First, clear understanding: the AI provided plain-language explanations of the medical terminology and findings, allowing for immediate understanding of the situation.

Second, emotional processing: having time to digest the information privately at home enabled both patient and caregiver to process the implications before the specialist visit. It is well established that receiving bad news, particularly news about a serious or life-altering medical condition, can significantly impair a patient’s ability to recall information presented during that conversation [10].

Third, recall: In a study examining patient recall of information after a neurosurgery consult, 44 patients were able to remember correctly 24.8 % of medical information on the next day after consultation by a neurosurgeon on average. Findings showed a correlation between patient mood and level of recall: a higher level of anxiety or depression before surgery was associated with less memory of medical information by patients [11].

In MBS’s words:

When we read the interpretation of my husband’s MRI test from our AI questions, we learned that he likely had to have neurosurgery, so that sent us reeling. It took us each several days to process that. I actually cried on and off for two days thinking about how that would impact my husband because he’s such an active guy. If we were in the doctor’s office when we learned about the need for spinal surgery, we would have been too shell-shocked to process much of the medical information.

Before we read the AI results, we didn’t even know what kind of doctor we should see as a next step. From the AI results, we knew that he should see a neurosurgeon, and we were then able to talk to people who had personal experience with the back surgeries he probably needed, and were able to get surgeon recommendations. Those conversations were very comforting. Without AI, our timeline for doing those things would have taken weeks versus days because we were able to take action right away.

#### Step 2: Using AI to Understand Treatment Options Enabled Faster, More Confident Action

After MBS received the results to her initial query (sometimes called an “AI conversation”), she performed a second query, doing a deeper dive using new questions to understand more about two of the possible surgical treatments: a laminectomy and a spinal fusion. Armed with new information, MBS was able to move forward with greater confidence. This helped them plan for possible surgery, prepare their home for recovery, and decide whether to postpone an upcoming trip.

In my case, I used the result of my first query to formulate my second set of questions to learn more about two of the possible surgeries and recoveries. The ability to keep doing research, jumping off of new information, allowed both of us to digest information in smaller pieces, and really drive the information gathering process according to the questions that were top of mind at the time. I used the following questions for each of the possible surgeries:

1. Can you tell me more about laminectomies?
2. Can you describe the surgery?
3. What is the recovery time?
4. What are the risks?
5. How soon before people typically go back to their normal lives?

#### Step 3: Using AI to Create a 1-Page Summary Supports Faster and More Efficient Care Navigation

In terms of next steps, the family knew they needed to communicate with their primary care physician, a neurosurgeon, a physical therapist, and the administrative staff to start. In order to communicate the complexities of the situation, MBS thought a 1-page summary would be helpful, especially to inform them about her significant concern about RL’s rapid progression. MBS went to her husband’s patient portal to view his health summary but found it insufficiently presented the information pertinent to his situation; she then used ChatGPT to create a single-page summary using the following approach:

I basically created a document in Microsoft Word as a place to gather information across multiple documents: my husband’s health portal, his radiology portal, and my own notes. I cut and pasted information from a variety of sources into the Microsoft Word document.

[MBS]

This information included demographics, health status, test results, symptoms, and observations over time. Details are shown in Figure 1.

After all of this information was collected in the Microsoft Word document, MBS was careful to remove the identifying information, such as name, address, and contact information in anticipation of cutting and pasting the information in ChatGPT. ChatGPT was then asked to create a 1-page summary of the above information, and be sure to include the complete progression of symptoms.

One of the strengths of ChatGPT is that it is great at organizing messy information—both visually and from an organizational standpoint. ChatGPT generated a nice, clean summary, with clear headings to organize the information. I took that 1-page summary and put it in a new Microsoft Word document, then added my husband’s name, our address, and contact information back into the document and saved it as a PDF document so it would be easy for me to share in text, email, and faxes.

[MBS]

With the help of AI, multiple documents and reports were pulled together into a single, streamlined summary containing all key details. This made it easier to share the information and provided clinicians and administrative staff a quick “at-a-glance” view of RL’s case without the need to search through portals, test results, and scattered files.

The family shared the summaries with a handful of people along their journey—the primary care physician to request a referral, 2 physical therapists, their insurance company, a hospital access nurse who facilitated neurosurgery scheduling, and the surgeon who requested to review the case to determine if a consult would be scheduled.

MBS explained:

I believe this saved us significant time in navigating the health care system. It was also really important to me that the neurosurgeon heard our direct account of how fast my husband’s progression happened, rather than having that information filtered through a third party. I’m confident that having clear documentation of my husband’s symptoms and rapid decline helped move his surgery forward more quickly.

#### Step 4: Generating Questions With AI to Prepare for a Surgical Appointment

Once the family scheduled a consultation with a neurosurgeon, they returned to ChatGPT, asking: What questions should I ask a neurosurgeon during a consultation based on this 1-page summary and MRI test result?

MBS then reviewed the AI-generated questions and went about editing the list of questions; “I modified the list of questions, adding several about upcoming travel and capacity to walk, exercise, and golf.” She prioritized the most and least important questions in case the visit time became limited. MBS felt that ChatGPT suggested questions that she would not have thought about on her own. The family felt more prepared and equipped with a thorough list.

We also felt much better prepared because we had a deep understanding of the implications of my husband’s test results and possible surgeries, and weren’t burdened with the anxiety of just hearing bad news for the first time about him needing neurosurgery. We were much more educated, having done our deep dive on the two possible surgeries, so we didn’t have to waste time having the neurosurgeon educate us on those. It was a much more productive doctor’s appointment than we would have had otherwise.

[MBS]

## Lessons Learned in Patient and Caregiver Use of AI

This case revealed several practical insights about how generative AI can support patients and caregivers in real time. The following lessons highlight what worked, what to watch for, and how others might benefit from similar and/or additional uses.

### Balancing AI’s Potential With Its Risks

**Figure 2.**
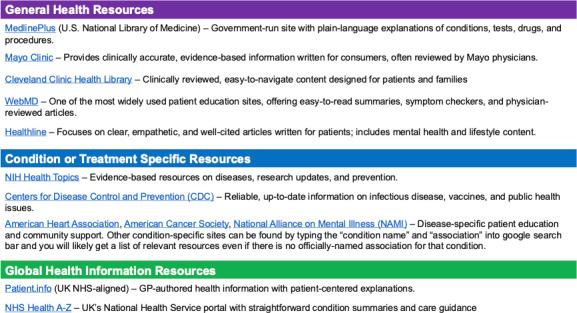
Some of the top sources that can be used by patients to verify medical information. NIH: National Institutes of Health; NHS: National Health Service; GP: general practitioner.

Once patients gain access to and understand their medical documentation, AI can become a powerful tool to research conditions and explore treatment options. A research or inquiry phase requires a careful balance between gathering comprehensive information and maintaining awareness of AI limitations. While AI can aggregate and explain medical information rapidly, all findings should be verified with other sources and with clinicians. MBS explains how she cross-checked AI results, all of which helped validate the output:

- Try more than one AI tool: it can help to compare answers. MBS asked both ChatGPT and Claude (Anthropic) the same question about MRI results to see if they gave similar responses.
- Look for sources: many AI tools provide links to sources at the end of their answers. Click on them to see where the information came from. If no sources are shown, you can ask the AI tool to provide them.
- Be cautious with vague sources: if a source looks unclear or takes you to a website that does not seem professional, double-check the information on a more reliable site (Figure 2).
- Check across websites: type the same keyword into the search bar on 2 or more trusted health websites (Figure 2). If what you find matches the AI’s response, it’s likely accurate.
- Ask someone knowledgeable: If possible, share the AI’s answer with a friend, family member, or health care professional who has medical experience. They might be able to confirm whether it makes sense.
- Use “research” features: some tools, like ChatGPT or Claude, offer deeper research modes. These can generate results that are verified and offer citations, but the process takes longer. Each LLM has a different method for using Deep Research Mode, so it is best to ask the LLM for directions on how to access it.

## Some Reputable Sources and Why Validation Matters

In MBS’s case, AI tools were helpful at multiple points of their journey—to translate complex medical data, summarize information, and guide them to questions for the doctor. Yet AI can produce mistakes or hallucinations—plausible-sounding answers that are factually incorrect or incomplete. These can be tricky because they seem so realistic. Since health decisions depend upon accurate information, it is essential to double-check AI output against trusted sources and, whenever possible, confirm with a health professional. While there are scores of reputable health-related websites, the list above is a short list of reliable, patient-friendly websites that can be used to verify information and lower the risk of acting on misinformation.

## Using LLMs to Understand Medical Documents

**Figure 3.**
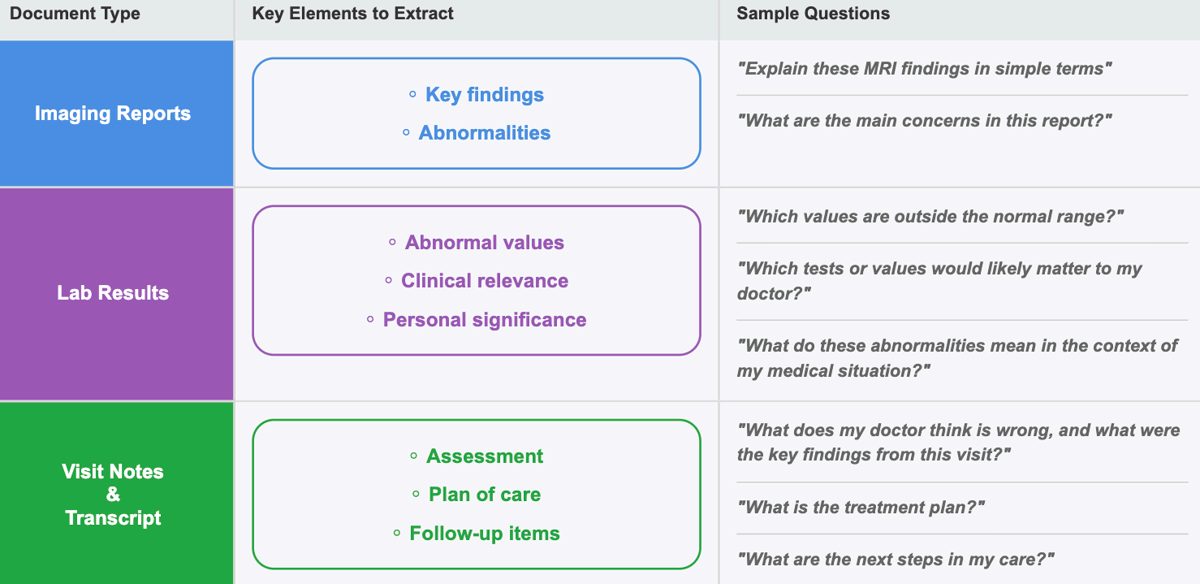
Strategies for patients and caregivers to use artificial intelligence to understand medical documentation. MRI: magnetic resonance imaging.

One of the most immediate applications of AI in patient care is the translation of complex medical documentation into understandable language. The disconnect between information access and understanding can lead to anxiety, delays in care, and missed opportunities for informed decision-making. Figure 3 shows some document types, elements to be explored within them, and sample questions one can use to reach those elements using tools like ChatGPT or other LLMs.

Transforming complex medical language into accessible information is powerfully illustrated in MBS’s case, but AI translation extends beyond imaging reports. Laboratory results, often presented as a series of numbers and technical terms, can be transformed into meaningful insights. When reviewing a comprehensive metabolic panel, for example, AI can explain not only what each value means, but also the relevance and gravity of any abnormalities in the context of a patient’s condition. With these explanations, patients can use AI to craft thoughtful questions to ask their health provider.

Some patients can become anxious when seeing test results on their own outside the doctor’s office. In a survey conducted by the University of Colorado of over 8000 patients, 96% of respondents preferred immediate delivery of test results—even before discussing results with their clinician. The authors noted, however, that about 8% of the respondents were worried when receiving the information [12]. Therefore, it is important to recognize that using AI to gain understanding of test results—without context from a clinician—can increase worry in some people.

Clinical notes, traditionally written in medical shorthand and jargon, can become more accessible to patients through AI interpretation, assuming AI is specifically asked to transform clinical notes into patient-friendly language. This helps patients better understand treatment options and plan next steps. Additionally, it allows patients to identify and track critical action items that might otherwise be lost in technical language.

Though a note of caution—when submitting health information such as test results to an AI tool, users should always remove all identifying details such as name, phone number, patient ID, birthdate, social security number, address, etc. This protects privacy and helps ensure personal health information is not exposed or misused. It is also recommended to remove the names of your health professionals.

## Diagnosis and Treatment Research is Valuable but Requires Understanding AI Shortcomings

Once patients understand their medical documentation, AI can become a powerful tool for researching conditions and exploring treatment options. This research phase requires a careful balance between gathering comprehensive information and maintaining awareness of AI’s limitations.

In MBS’s case, understanding the implications of various surgical options before the specialist consultation allowed for a more productive discussion focused on specific concerns rather than basic explanations. AI can help break down:

- Potential treatment approaches and their rationales.
- Common risks and benefits of each option and the type of specialist that can provide treatment.
- Typical recovery timelines and requirements.
- Questions that would be important to discuss with specialists.

As previously discussed, while AI can aggregate and explain medical information rapidly, all findings should be validated with reputable sources and ideally with health professionals.

**Figure 4.**
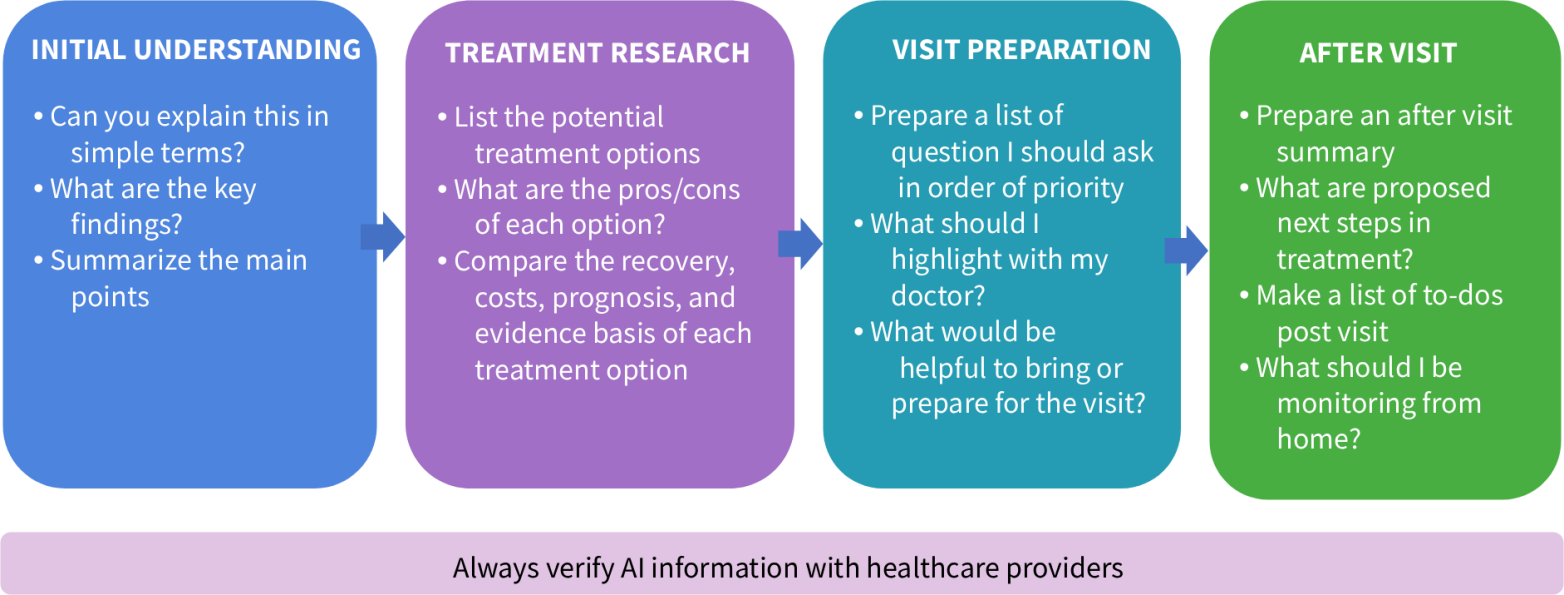
Sample prompts and queries to research patient health data and medical information.

Figure 4 shows examples of research that patients can conduct using ChatGPT or other LLMs, with some sample questions showcasing several areas where patients might find AI helpful in researching their own medical conditions.

Keep in mind that sophisticated AI skills are not necessarily needed to get valuable information. Users can simply ask questions as if sitting in a doctor’s office, and continue asking as you learn from the AI output. AI users can also specify how results are desired, for example—as a bulleted list, no more than 1 page, in Spanish or other languages, using only academic research reports, or in patient-friendly language, etc.

A major advancement in consumer health is the ability for patients to track vitals and symptoms using a variety of apps. AI tools can help organize symptoms into timelines and summaries, identify known medical conditions, and suggest possible diagnoses ranked by likelihood. AI can connect the dots—highlight patterns, surface insights, and suggest questions patients may want to ask their doctor. This allows patients to go beyond simple internet searches on a single symptom and better understand what individual symptoms, groups of symptoms, or changes in symptoms over time might mean. Again, any AI-generated results need to be validated against reliable medical sources and a clinician.

Treatment plans, particularly for complex conditions requiring multiple interventions, can be broken down into manageable steps. Complex treatment—such as daily wound care, intricate medication dosing schedules, or tracking specific symptoms—can be separated into clear, actionable daily checklists that improve adherence and outcomes. In MBS’s case, a systematic approach was used and resulted in a compressed timeline between receiving medical information and taking informed action.

## Bringing Patient and Caregiver Voice to Clinicians Across Care Teams

Navigating today’s complex health care system is a significant challenge for families, particularly during health crises. AI tools can help patients manage this complexity by organizing information, preparing for appointments, and coordinating between providers.

The value of using AI for health system navigation was evident in MBS’s case when coordinating care across multiple specialists. What traditionally may require multiple, lengthy phone calls with repeated explanations could be streamlined: a single concise summary can be shared with the primary care and specialist physicians and used to facilitate referrals and second opinion consultations.

Using AI-generated text to support patient verbal explanations can offer consistent communication across all clinicians while preserving the authentic voice of patients and caregivers. This approach is especially valuable when a patient needs to explain complex information—often involving multiple symptoms over time. AI text summaries of patient-reported information can help ensure that patients and caregivers do not lose their personal voice or lived experience and can communicate accurately and efficiently. By reducing reliance on patients' memory “in the moment” or need for verbal explanations to be repeated for each provider, AI helps prevent important details from being overlooked.

## Visit Preparation Can Optimize Time With Doctors

Visit preparation represents another area where AI can significantly improve patient care. When patients arrive at visits with a basic understanding of their condition and well-researched questions, clinicians can focus on the more complex and personalized aspects of care. Patient and symptom summaries can condense a lot of information into “quick takeaway” documents that make the most of short appointment times. As MBS’s case demonstrates, patients who arrive better informed and confident can have more meaningful conversations. Some examples of preparation include:

- Summarizing your current health situation, or updates since last visit.
- Organizing test results, summary of symptoms.
- Developing specific, informed questions.
- Preparing clear descriptions of new concerns.

## Some AI Tools for Patient Empowerment in Health Care

**Figure 5.**
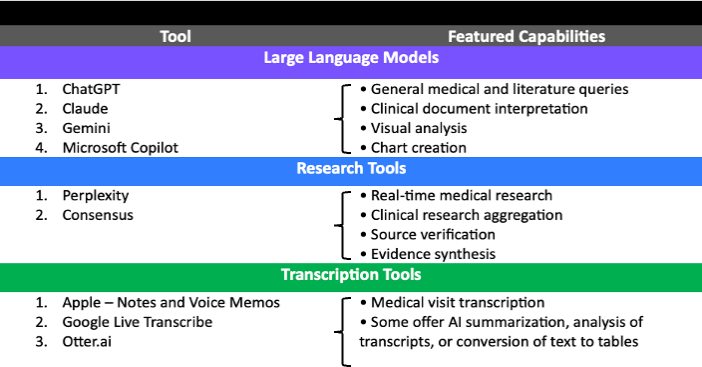
Sample artificial intelligence tools for patient and caregiver empowerment that may help a health care journey.

The effective use of AI in health care requires understanding the diverse landscape of available tools and selecting the right one for each task. While many AI tools exist, they broadly fall into 3 main categories, each with different strengths. Examples of several tools are shown in Figure 5.

### Large Language Models

The foundation of patient AI use typically starts with general-purpose AI tools like ChatGPT, Claude, or Google’s Gemini. These serve as versatile assistants capable of handling a wide range of medical information needs, functioning as digital “Swiss Army knives” for health care applications. LLMs can perform tasks ranging from basic medical terminology translation to visual analysis of skin conditions through image recognition features. LLMs can review and organize even complicated medical texts, including research papers and clinical guidelines, into a more patient-friendly language.

Part of what makes LLMs so powerful is that LLMs are “trained on” (have access to) massive amounts of information—the equivalent of reading millions of books or the entire internet many times over. This includes publicly available data such as websites, articles, news, blogs, educational resources, and public health information from sources like the Centers for Disease Control and Prevention, World Health Organization, and the National Institutes of Health, as well as open-access scientific journals. Some LLMs also include licensed content**,** including textbooks, databases, or large datasets. LLMs do not have access to private health records, real-time clinical databases, or most subscription-based information.

LLMs are also trained on general medical knowledge sources (not patient-specific), including medical textbooks, clinical guidelines, reference materials, and research abstracts or summaries from medical databases. Using this vast amount of information, LLMs can connect related pieces (eg, 4 different symptoms that might not seem related) and present them in clear, easy-to-understand ways. By contrast, humans have experiential knowledge, intuition, clinical judgment, and real-world pattern recognition, whereas LLMs have statistical patterns from text, but no direct experience. Both can be valuable, and even more so when combined.

It is important to emphasize that AI output does not constitute a medical diagnosis or should not drive clinical decision-making. Medical knowledge evolves rapidly, and there can be lags between new research and what is reflected in LLM training data. The ability to “link four different symptoms” is pattern recognition based on an LLM’s training data, but not clinical reasoning and view of the full patient background that a health care provider would have.

### Research Tools

When patients need to delve deeper into medical literature or find specific clinical information, specialized research tools become invaluable. Perplexity AI stands out for its ability to find and synthesize current medical information, guidelines, and reviews in real time. Its advanced search capabilities allow patients to specifically target medical journals for scientific evidence, or alternatively, explore patient communities on platforms like Reddit for valuable real-world experiences and insights.

### Transcription Tools

Recording conversations can help many patients to remember all of the fine details from doctor’s appointments. Transcription capabilities are available, whether through native apps like Apple’s voice memo transcription or dedicated tools, which enable patients to record and transcribe medical consultations. Many health systems now use what is referred to as “ambient documentation” technology that records visits, and these transcripts are often available upon request. These transcriptions can then be input into LLMs to understand health care providers’ rationale and planned next steps, giving AI comprehensive data to better serve patient needs.

It is important to obtain a clinician’s consent before recording a medical visit, just as they would need patient consent. Often required by law, this helps maintain trust, respect, and transparency between patient and provider [13].

## Future Directions

After reviewing what has worked and what to be mindful of in terms of how patients use AI, the next step is to consider how AI can be improved and better support patients in the future. The evolution of AI in health continues at a rapid pace. Going forward, with emerging technologies and changing health care dynamics, there are exciting possibilities and important considerations for patient empowerment through AI. Current publicly available AI applications operate independently of health care organizations; AI integrating with health care systems represents perhaps the most promising frontier.

When MBS wanted to use AI to translate MRI results, she needed to manually copy text from the patient portal into ChatGPT. Imagine patient portals equipped with AI assistants that can immediately help interpret new test results or explain changes in treatment plans. These applications could achieve levels of privacy and security of existing health care platforms, while adding the immediate comprehension support that patients need. A more streamlined future might include:

- “Explain This” buttons next to medical terms in patient portals.
- AI assistants that maintain context across multiple health documents or care providers.
- Tools that help prepare visit summaries by combining AI analysis of both clinical notes and visit recordings.
- Allowing electronic health record data to be exported and deidentified for use with other AI tools.
- Systems that suggest relevant questions based on upcoming appointment types.

### Visual Interpretation Tools

Visual interpretation tools also show particular promise. While current AI applications excel at text interpretation, emerging technologies demonstrate increasing capability in analyzing medical imaging. Future systems might help patients better understand their X-rays, MRIs, or computed tomography scans through:

- Interactive 3D visualizations of findings.
- Comparative analyses with previous imaging.
- Plain-language explanations of visual changes.
- Preparation guides for upcoming imaging procedures.

### Personalization

Personalization represents another crucial area of development. Future AI systems will likely become more adept at tailoring their support based on:

- Individual language preferences.
- Personal medical history.
- Individual health literacy levels.
- Learning style preferences (eg, text, video, and photos).
- Specific medical conditions.

This personalization extends beyond mere language adjustments to encompass comprehensive adaptation of information delivery and support strategies.

### Diagnostic Support

A significant portion of medical research never reaches clinicians or their patients, and translating new evidence into routine practice is often estimated to take about 17 years [14]. AI can analyze large medical datasets, detect subtle patterns, and link symptoms that might otherwise seem unrelated, making it a powerful tool for diagnostic support [15,16]. Although AI lacks access to every piece of medical data, it can draw on extensive, diverse sources to provide insights directly to patients. Like humans, AI can make mistakes, but it offers an important advantage: generating a list of potential explanations or conditions for patients and clinicians to review and validate together.

### Supporting Underserved Communities

Supporting underserved communities with AI can assist them by providing health information around the clock and in multiple languages, even when clinics are closed. For example, a patient could ask the same questions MBS used but add, “Translate this into Spanish.” As shown in MBS’s case, AI can translate complex medical reports into plain-language summaries that increase patient understanding [17] and deliver information in ways that overcome language barriers and limited clinic hours.

Additionally, certain populations, such as older people, may be more comfortable interacting via speech, which is a feature in many LLMs, as some individuals may find the dexterity involved with typing on small keypads on smartphones challenging [18].

### Overcoming Barriers

There are barriers that some patients may face in using large language models, including subscription costs, limited digital literacy, and a lack of knowledge about how to work with AI tools. Public libraries, senior centers, and community organizations—such as local councils on aging—can help bridge these gaps by offering free internet access, technology training, and support to help build skills.

It’s important that these advancement suggestions above be balanced against important considerations. Privacy and security concerns will require ongoing attention as AI systems become more integrated with personal health information. The development of robust security protocols, clear ethical guidelines, and strategies to detect and mitigate data bias must proceed in parallel with technological advancement.

Perhaps most importantly, future developments must maintain focus on a fundamental goal: enhancing patient care and outcomes. Technology should serve patient needs with development priorities shaped by actual patient experiences and health care requirements.

As we look ahead, several key principles should guide the evolution of AI in patient care:

1. Improve patient outcomes and experiences.
2. Enhance understanding and engagement.
3. Maintain human connection in care.
4. Ensure equity, access, and privacy.
5. Support clinical judgment—and cause no harm.

The future of AI in patient care holds tremendous promise, but realizing this potential requires thoughtful development and implementation. Progress should be measured less by technological innovation and more by meaningful improvements in patient outcomes and experiences. Further study is needed to better understand the impact of these emerging tools on both. As MBS’s case demonstrated, the goal is not to revolutionize health care but to make it more accessible, understandable, and effective for patients navigating their health journeys.

## Conclusion

This case study shows how integrating AI tools into patient care can greatly enhance the patient and caregiver experience. ChatGPT helped turn a stressful period into one of clearer understanding and active engagement. Such tools can increase patient understanding, improve communication with clinicians, support treatment research, ease emotional strain, and shorten the care timeline.

As health care evolves, AI has the potential to support patients across their health care journey, working alongside providers. Properly implemented, AI can empower patients to participate more effectively in their care, provided its output is validated by trusted sources. To succeed, these tools must be designed with patients and caregivers at the center, addressing their distinct challenges and needs.

## Abbreviations

AI: artificial intelligence
LLM: large language model
MRI: magnetic resonance imaging
